# Toll-Like Receptor 4 Modulates Small Intestine Neuromuscular Function through Nitrergic and Purinergic Pathways

**DOI:** 10.3389/fphar.2017.00350

**Published:** 2017-06-08

**Authors:** Valentina Caputi, Ilaria Marsilio, Silvia Cerantola, Mona Roozfarakh, Isabella Lante, Francesca Galuppini, Massimo Rugge, Eleonora Napoli, Cecilia Giulivi, Genny Orso, Maria Cecilia Giron

**Affiliations:** ^1^Department of Pharmaceutical and Pharmacological Sciences, School of Medicine, University of PadovaPadova, Italy; ^2^San Camillo HospitalTreviso, Italy; ^3^Medway School of Pharmacy, Universities of Kent and Greenwich at MedwayKent, United Kingdom; ^4^Department of Medicine, University of PadovaPadova, Italy; ^5^Department of Molecular Biosciences, School of Veterinary Medicine, University of California, Davis, DavisCA, United States; ^6^Medical Investigation of Neurodevelopmental Disorders Institute (M.I.N.D.), University of California, Davis, SacramentoCA, United States; ^7^IRCCS “E. Medea" Bosisio PariniLecco, Italy

**Keywords:** toll-like receptor 4, enteric nervous system, small bowel, intestinal motility, intestinal transit, innate immunity, gut microbiota, knockout mice

## Abstract

**Objective:** Toll-like receptors (TLRs) play a pivotal role in the homeostatic microflora-host crosstalk. TLR4-mediated modulation of both motility and enteric neuronal survival has been reported mainly for colon with limited information on the role of TLR4 in tuning structural and functional integrity of enteric nervous system (ENS) and in controlling small bowel motility.

**Methods:** Male TLR4 knockout (TLR4^-/-^, 9 ± 1 weeks old) and sex- and age-matched wild-type (WT) C57BL/6J mice were used for the experiments. Alterations in ENS morphology and neurochemical code were assessed by immunohistochemistry whereas neuromuscular function was evaluated by isometric mechanical activity of ileal preparations following receptor and non-receptor-mediated stimuli and by gastrointestinal transit.

**Results:** The absence of TLR4 induced gliosis and reduced the total number of neurons, mainly nNOS^+^ neurons, in ileal myenteric plexus. Furthermore, a lower cholinergic excitatory response with an increased inhibitory neurotransmission was found together with a delayed gastrointestinal transit. These changes were dependent on increased ileal non-adrenergic non-cholinergic (NANC) relaxations mediated by a complex neuronal-glia signaling constituted by P2X7 and P2Y1 receptors, and NO produced by nNOS and iNOS.

**Conclusion:** We provide novel evidence that TLR4 signaling is involved in the fine-tuning of P2 receptors controlling ileal contractility, ENS cell distribution, and inhibitory NANC neurotransmission via the combined action of NO and adenosine-5′-triphosphate (ATP). For the first time, this study implicates TLR4 at regulating the crosstalk between glia and neurons in small intestine and helps to define its role in gastrointestinal motor abnormalities during dysbiosis.

## Introduction

The functional link between endogenous gut microbes and their host regulates the balance between commensalism and parasitism on the microbial side, and between infection and resistance on the host side. Although this complex bidirectional communication is still not fully understood, it may be involved in the onset of gastrointestinal functional disorders (GFD), such as irritable bowel syndrome (IBS) (Barbara et al., 2016).

Commensal enteric microbiota efficiently influences innate defenses by preventing pathogenic bacteria from crossing the mucosal barrier (Kamada et al., 2013). However, this delicate balance can be perturbed by several factors (e.g., stress, drugs, and inflammation) leading to GFD as a result of a vicious cycle in which host dysfunctions affect microflora environment leading to dysbiosis and vice versa (Rakoff-Nahoum et al., 2004; Lee and Lee, 2014). These disorders are characterized by intestinal barrier breakdown, bacterial translocation and changes in motility (Bischoff et al., 2014), and appear to be driven by abnormal responses to microbiota-derived molecules via stimulation of Toll-like receptors (TLRs) (Rakoff-Nahoum et al., 2004; Barbara et al., 2016).

The finding of TLRs expressed in both central and enteric nervous systems (CNS and ENS) (Barajon et al., 2009; Okun et al., 2011), acting as key sensors not only for damage-associated molecular patterns but also for physiological factors, strongly extends the impact of TLRs in the nervous system beyond their role in controlling host immune responses (Okun et al., 2011). In other words, in absence of any underlying immune response, the location in the nervous system of TLRs and their activation by endogenous ligands underlies their role as key players in regulating neurodevelopment and neuroplasticity (Aravalli et al., 2007; Okun et al., 2011). Among all TLRs, TLR4 is the best characterized pathogen-recognition receptor and recently recognized to modulate ENS phenotype and function (Anitha et al., 2012). We recently showed the expression of TLR4 mRNA transcripts in freshly isolated smooth muscle cells and enteric glial cells (EGCs) but not in resident macrophages and dendritic cells (Brun et al., 2015). *In vivo*, under steady-state conditions, TLR4 is generally absent or detected in very small amounts in intestinal epithelial cells and in immune cells located in the subepithelial lamina propria, in order to avoid inappropriate activation despite the omnipresent microbiota (Cario, 2010).

Several studies advocate for a role of TLRs in ENS homeostasis (Kabouridis and Pachnis, 2015). TLR2^-/-^ mice show disrupted ENS structural and functional integrity, similar to that observed in antibiotic-induced microbiota depleted wild-type mice (Brun et al., 2013). Germ-free, antibiotic-treated mice and TLR4 signaling-defective (*Tlr4^Lps-d^)* mice show similar colonic dysmotility and fewer nNOS^+^ neurons (Anitha et al., 2012). Furthermore, treatment with low lipopolysaccharide (LPS) levels promotes the survival of cultured enteric neurons in an NF-κB–dependent mechanism (Anitha et al., 2012).

Based on these evidences, we sought to characterize the role of TLR4 in tuning structural and functional integrity of ENS and in controlling small bowel contractility for identifying the signaling pathways involved in neuroimmune cross talk, hopefully translatable into novel therapeutic strategies for patients with GFD.

## Materials and Methods

### Animals

Male TLR4^-/-^ (B6.B10ScN-Tlr4^lps-del^/JthJ; 9 ± 1 weeks old) and sex- and age-matched wild-type (WT) C57BL/6J mice (Jackson Laboratories, Bar Harbor, ME, United States) were housed in individually ventilated cages (IVC) at the animal facility of the Department of Pharmaceutical and Pharmacological Sciences, University of Padova. To normalize gut microbiota, mice colonies from both groups were housed in the same room and generally in the same cages, and maintained by the same personnel. All animals were specific pathogen-free and given standard chow diet and tap water *ad libitum*. All experimental protocols were approved by the Animal Care and Use Ethics Committee, University of Padova and Italian Ministry of Health (authorization number: 1142/2015-PR) and are reported in compliance with ARRIVE guidelines (McGrath and Lilley, 2015).

### Histology

Ileal specimens, fixed in 10% buffered formalin, embedded in paraffin and cut into 4 μm-sections were stained with haematoxylin and eosin (H&E) (Dall’Olmo et al., 2014). Ten slides for each genotype were analyzed blindly.

### Intestinal Paracellular Permeability

Intestinal paracellular permeability was assessed as previously described (Aubé et al., 2006). Briefly, WT and TLR4^-/-^ mice were gavaged orally with absorbable fluorescein isothiocyanate (FITC)-dextran (4 kDa molecular weight; 200 μl, 600 mg/kg body weight). Preliminary experiments at various time points (0, 0.5, 1, 2, 4, and 6 h) showed that the appearance in blood of low molecular weight FITC-dextran peaked at 4 h following oral administration in WT and TLR4^-/-^ mice. After 4 h, FITC-dextran serum concentration was determined using a fluorimeter (PerkinElmer, Milan, Italy) at 490/530 nm.

### Gastrointestinal Transit

WT and TLR4^-/-^ mice were gavaged orally with 70-kDa FITC-dextran (25 mg/ml in 0.9% saline solution). Preliminary experiments at various time points (0, 30, 45, 60, 75, and 90 min) showed that the distribution of high molecular weight FITC-dextran peaked at 30 min in the ileum following oral administration in WT and TLR4^-/-^ mice. After 30 min, the stomach and caecum were examined separately while small intestine and colon were divided into 10 and 3 comparable length-segments, respectively. Gastrointestinal transit was determined using the intestinal geometric center of FITC-dextran distribution throughout the intestine as described previously (Aubé et al., 2006; Brun et al., 2013). Gastric emptying was determined by percentage of fluorescent probe that emptied the stomach (Aubé et al., 2006).

### Pellet Frequency and Fecal Water Content

Fecal pellet output was assessed daily as previously described. Mice were observed for 60 min. Fecal pellet numbers, fecal wet weights and dry weights were determined. The difference between wet and dry weights was used to calculate fecal water content (McQuade et al., 2016).

### *In Vitro* Contractility Studies

Contractility studies were performed as previously described (Giron et al., 2008; Brun et al., 2013; Zoppellaro et al., 2013). Briefly, 1-cm longitudinal segments from the distal ileum were mounted in 10-mL-organ baths equilibrated for 30 min in Krebs solution (37°C). Changes in muscle tension were recorded by isometric transducers connected to a PowerLab4/30 system (ADInstruments, Oxford, United Kingdom). Carbachol (0.001–100 μM) and ADP (0.001–1 mM) dose-response curves were obtained cumulatively. Non-receptor-mediated contractile responses to 60 mM KCl were assessed. Neuronal-mediated contractions were analyzed following electrical field stimulation (EFS; 0–40 Hz; 40 V) in basal conditions or in non-adrenergic non-cholinergic (NANC) conditions, obtained by adding 1 μM guanethidine and 1 μM atropine to the organ bath. 10 Hz-EFS-mediated NANC responses were evaluated in presence of the pan-NOS inhibitor L-NAME (100 μM), the iNOS inhibitor 1400W (10 μM), the P1-purinoceptor antagonist theophylline (100 μM), the P2-purinoceptor antagonist suramin (100 μM), the P2Y1 receptor (P2Y1R) antagonist MRS2500 (1 μM) or the P2X7R antagonist A804598 (0.1 μM).

Ten hertz-EFS-mediated tachykininergic responses were recorded in NANC conditions with 100 μM L-NAME. Contractile responses were expressed as gram tension/gram dry tissue weight of ileal segments. Ileal relaxation was calculated as the percentage reversal of the initial gram tension/dry tissue weight, setting 100% inhibition as the maximum relaxation (Zizzo et al., 2003).

### Acetylcholine Esterase and NADPH-Diaphorase Biochemical Staining

Distal ileal 10 cm-segments were filled with fixative solution (4% paraformaldehyde in PBS) for 1 h at room temperature. Using a dissecting microscope, whole mount specimens of longitudinal muscle-myenteric plexus (LMMP) were prepared as previously described (De Mello et al., 2009; Brun et al., 2013). Briefly, LMMP preparations were gently stretched and pinned down on a wax support and subjected to acetylcholine esterase (AChE) or NADPH-diaphorase (NADPHd) biochemical staining (Anitha et al., 2006). Stained tissues mounted on glass slides were observed using a Leica DMI4000 B microscope (Leica Microsystems GmbH, Wetzlar, Germany). AChE^+^ myenteric fibers and NADPHd^+^ neuronal cells analysis was performed blindly by counting fibers or neurons in 10 randomly-chosen images per mouse (six animals/group), as previously described (De Mello et al., 2009).

### Immunohistochemistry

Longitudinal muscle-myenteric plexus whole mount preparations were gently stretched and pinned down on a wax support and permeabilized in PBT (PBS with 0.3% Triton X-100) and blocked with 2% bovine serum albumin (BSA) for 1 h at room temperature, as previously reported (Brun et al., 2013). Distal ileum (0.5 cm-segments) were frozen in optimal cutting temperature mounting medium (OCT), sectioned (7 μm-thick) with a cryostat (Leica CM 1850 UV, Milan, Italy) and then mounted onto Superfrost Plus slides. From each ileal specimen, 100 sequential 7 μm-cross-sections were cut on a cryostat and 6–8 sections were subjected to immunohistochemistry as previously described (Giron et al., 2008; Zoppellaro et al., 2013). LMMP whole mount preparations or ileal cryosections were then incubated overnight at room temperature with the following antibodies: chicken polyclonal anti-mouse glial fibrillary acidic protein (GFAP; 1:100; Abcam, Milan, Italy), rabbit polyclonal anti-human GFAP (1:200; Merck Millipore Corporation, Milan, Italy), mouse biotin-conjugated anti-human HuC/D (1:50; Thermo Fisher Scientific, Milan, Italy), rabbit polyclonal anti-mouse neuronal nitric oxide synthase (nNOS, 1:100; Thermo Fisher Scientific, Milan, Italy), rabbit polyclonal anti-human inducible NOS (iNOS; 1:50, Santa Cruz Biotechnology, Milan, Italy), rabbit polyclonal anti-human vasoactive intestinal peptide (VIP, 1:100; GenWay Biotech, Milan, Italy), rabbit polyclonal anti-human P2X7 receptor-ATTO-488 (1:100; Alomone labs, Jerusalem, Israel), rabbit polyclonal anti-human P2Y1 receptor (1:100; Alomone labs, Jerusalem, Israel), rabbit monoclonal anti-human S100β (1:100; Merck Millipore Corporation, Milan, Italy) and guinea pig polyclonal anti-mouse substance P (1:100; Abcam, Milan, Italy). Then cryosections or LMMP whole mount preparations were washed and incubated for 2 h at room temperature with the following secondary antibodies: Alexa Fluor 488-conjugated goat anti-chicken IgG (1:1000; Thermo Fisher Scientific, Milan, Italy), Alexa Fluor 488-conjugated goat anti-guinea pig IgG (1:500; Thermo Fisher Scientific, Milan, Italy), Alexa Fluor 488-conjugated goat anti-rabbit IgG (1:1000; Thermo Fisher Scientific, Milan, Italy), DyLight 649-conjugated goat anti-rabbit IgG (1:500, Jackson ImmunoResearch Laboratories, Milan, Italy) or Alexa Fluor 555-conjugated streptavidin (1:1000, Thermo Fisher Scientific, Milan, Italy). Nuclei were stained with TOTO-3 (1:500; Thermo Fisher Scientific, Milan, Italy) or with 4′,6-diamidino-2-phenylindole, dihydrochloride (DAPI) (1:1000; Thermo Fisher Scientific, Milan, Italy) added together with the secondary antibodies. After three washes, LMMP whole mount preparations or cryosections were mounted on glass slides using a Mowiol Mounting Medium (100-mM Tris-HCl (pH 8.5), 9% Mowiol 4–88, 25% glycerol and 0.1% DABCO). Negative controls were obtained by incubating sections with isotype-matched control antibodies at the same concentration as primary antibody and/or pre-incubating each antibody with the corresponding control peptide (final concentration as indicated by manufacturer’s instructions) (Brun et al., 2013).

### Confocal Image Acquisition and Analysis

Images were acquired using a Nikon D-Eclipse C1 confocal microscope (Nikon Instruments, Florence, Italy), equipped with an oil-immersion Nikon Plan-Apo 60×/1.4 objective or a low-magnification Nikon Plan Fluor 20×/0.5 objective. Five Z-series images (10 planes for ileum cryosections or 15 planes for LMMP whole mount preparations) of 1024 pixels × 1024 pixels were processed as maximum intensity projections. All microscope settings were kept constant for all images. Fluorescence intensity of S100β, substance P, P2X7 receptors, P2Y1 receptors and iNOS was assessed for each antigen in 20 images per mouse (*N* = 5 mice/group), as previously reported (Arqués et al., 2014). The obtained fluorescence values indicating the fluorescence intensity of each protein of interest (i.e., S100β, substance P, P2X7 receptors, P2Y1 receptors or iNOS) were normalized for the fluorescence intensity of its own TOTO-3 or DAPI and were reported as mean ± SEM, as previously described (Orso et al., 2005).

Fluorescence intensity of GFAP^+^ fibers was determined by applying the skeleton analysis method developed to quantify brain microglia morphology as previously described (Morrison et al., 2013). Briefly, for skeleton analysis, the maximum intensity projection of the GFAP^+^ channel was enhanced to image all enteric glial processes, followed by noise de-speckling to eliminate single-pixel background fluorescence. After converting the resulting images to binary, they were skeletonized using ImageJ software and then analyzed by the AnalyzeSkeleton plugin to determine the number of endpoints per frame and process length. These data were normalized to a total area of 13.14 mm^2^, obtained from 20 images per mouse (*N* = 5 mice/group) in order to assess changes in EGCs. Analyses of images and related fluorescence intensity were performed using ImageJ software (Fiji, version 1.51n).

In ileal LMMP whole mount preparations, total neuron population analysis was performed by counting HuC/D^+^ cells in 10 randomly-chosen images per mouse (*N* = 8 mice/group). The total number of HuC/D^+^ neurons was recorded in each image and normalized to the total area of 4.05 mm^2^. To evaluate the distribution of nitrergic and VIPergic neurons in ileal myenteric plexus, the number of nNOS^+^ and VIP^+^ enteric neurons was blindly counted in 10 randomly-chosen images per mouse (*N* = 8 mice/group) and normalized to the total area of 4.05 mm^2^.

### Chemicals

Unless otherwise specified, all chemicals were obtained from Sigma–Aldrich (Milan, Italy) and were of the highest commercially available analytical grade. OCT was purchased from Kaltek (Padua, Italy), PFA was from Electron Microscopy Sciences-Società Italiana Chimici (Rome, Italy), β-NADPH was obtained from Diagnostic Brokers Associated (Milan, Italy), and Triton-X-100 was from Applichem (Milan, Italy).

### Statistical Analysis

All data are expressed as mean ± SEM. Differences between the experimental groups were assessed using paired or unpaired Student’s *t*-test and one-way or two-way analysis of variance (ANOVA), followed by *post hoc* Bonferroni test. Data were analyzed using GraphPad Prism 3.03 (San Diego, CA, United States). The results were considered statistically significant at *p* < 0.05; “*N*” values indicate the number of animals.

## Results

### TLR4 Influences Ileal Morphology and ENS Architecture

**FIGURE 1:**
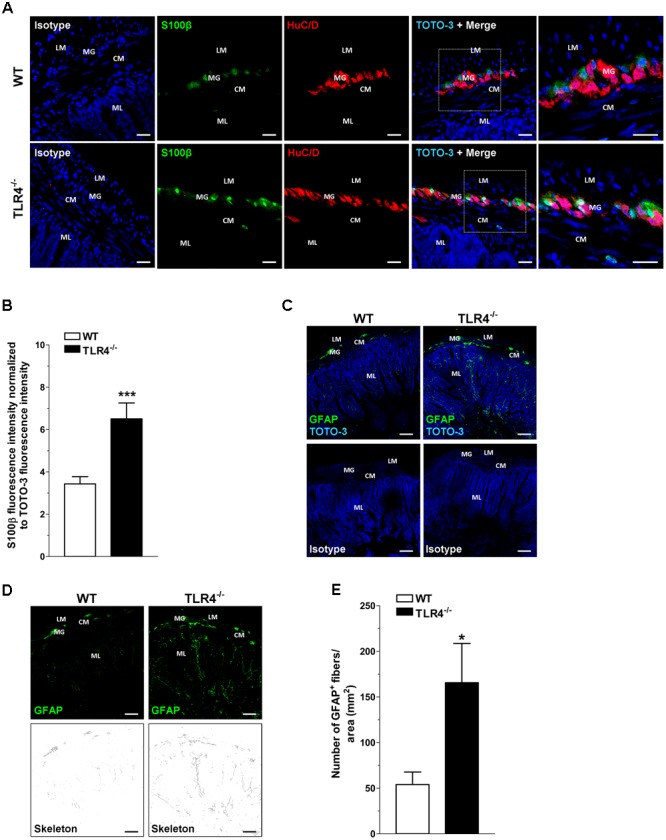
TLR4 deficiency alters glial phenotype. Representative confocal microphotographs **(A)** of HuC/D (red) and S100β (green) distribution in wild-type (WT) and TLR4^-/-^ cryosections and quantification **(B)** of S100β fluorescence intensity (bars = 22 μm). **(C)** Representative confocal microphotographs of glial fibrillary acidic protein (GFAP) distribution in WT and TLR4^-/-^ cryosections (bars = 75 μm). **(D)** Representative confocal maximum intensity projection of GFAP^+^ channel with the corresponding skeleton images. **(E)** Number of GFAP^+^ glial processes. Cell nuclei were stained with TOTO-3 (blue). ^∗^*P* < 0.05, ^∗∗∗^*P* < 0.001 vs. WT (*N* = 5 mice/group). LM, longitudinal muscle; CM, circular muscle; MG, myenteric ganglia; ML, mucosal layer.

Since TLR4 expression is required for normal growth (including villus height) of the small intestine (Riehl et al., 2015), we sought to determine whether the absence of TLR4 influences structural architecture by examining hematoxylin and eosin-stained sections. In agreement with previous findings (Riehl et al., 2015), TLR4^-/-^ ileal morphology was comparable to WT, except for villi height, which was significantly diminished (349 ± 9 μm in WT mice vs. 306 ± 15 μm in TLR4^-/-^ mice; *N* = 20 animals/group). No significant differences in serum levels of absorbable FITC-dextran were found between TLR4^-/-^ and WT mice (0.42 ± 0.1 μg/ml and 0.32 ± 0.1 μg/ml, respectively; *N* = 12 animals/group), to indicate no alterations in intestinal permeability. Considering that TLR4 is expressed in ENS (Rumio et al., 2006; Barajon et al., 2009), the impact of TLR4 absence on ENS integrity was evaluated by immunohistochemistry. In ileal cryosections, a 1.84-fold increase in the immunoreactivity of the glial marker S100β was found in TLR4^-/-^ myenteric plexus (**Figures 1A,B**). These increases in S100β immunoreactivity were associated to a 3.1-fold increase in process length of GFAP^+^ gliofilaments in TLR4^-/-^ ENS (**Figures 1C–E**).

### Absence of TLR4 Impairs Gastrointestinal Motility

Previous studies have shown a reduced gastrointestinal transit 4 h after non-absorbable FITC-dextran administration in C3H/HeJ mice with a spontaneous point mutation in *Tlr4* gene (*Tlr4^Lps-d^*) (Anitha et al., 2012). We hypothesized that the same functional impairment could be present in TLR4^-/-^ mice (B6.B10ScN-Tlr4^lps-del^), which are homozygous for a null mutation of *Tlr4* gene.

**FIGURE 2:**
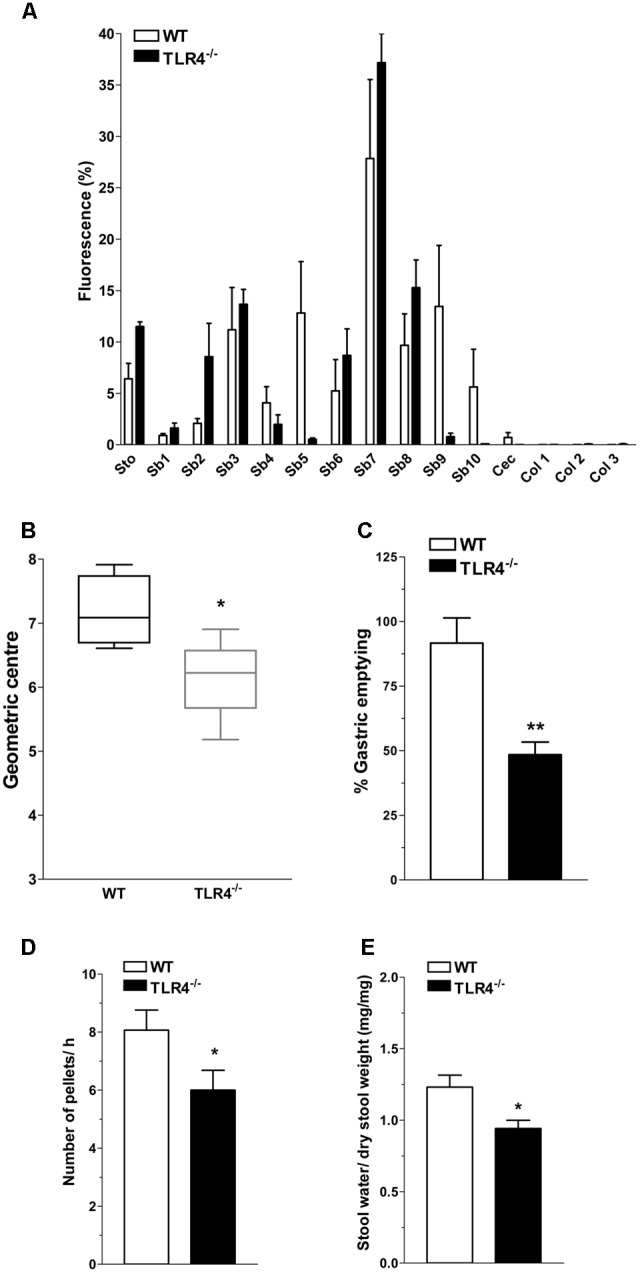
TLR4 signaling influences gastrointestinal transit and gastric emptying. **(A)** Percentage of non-absorbable fluorescein isothiocyanate (FITC)-dextran distribution along the gastrointestinal tract (stomach, Sto; small bowel, Sb 1–10; caecum, Cec; and colon, Col 1–3), **(B)** geometric center, **(C)** percentage of gastric emptying, **(D)** pellet frequency per hour, **(E)** fecal water content in WT and TLR4^-/-^ mice. ^∗^*P* < 0.05, ^∗∗^*P* < 0.01 vs. WT (*N* = 12 mice/group).

In WT mice, the non-absorbable FITC-dextran transited through the gastrointestinal tract over a 30-min period and localized at the terminal ileum (**Figure 2A**). Conversely, a significant reduction of geometric center and gastric emptying was observed in TLR4^-/-^ mice (**Figures 2B,C**). The number of fecal pellets/hour and stool water content were significantly lower in TLR4^-/-^ mice compared to WT mice (**Figures 2D,E**).

### TLR4 Deficiency Affects Excitatory Neurotransmission

**FIGURE 3:**
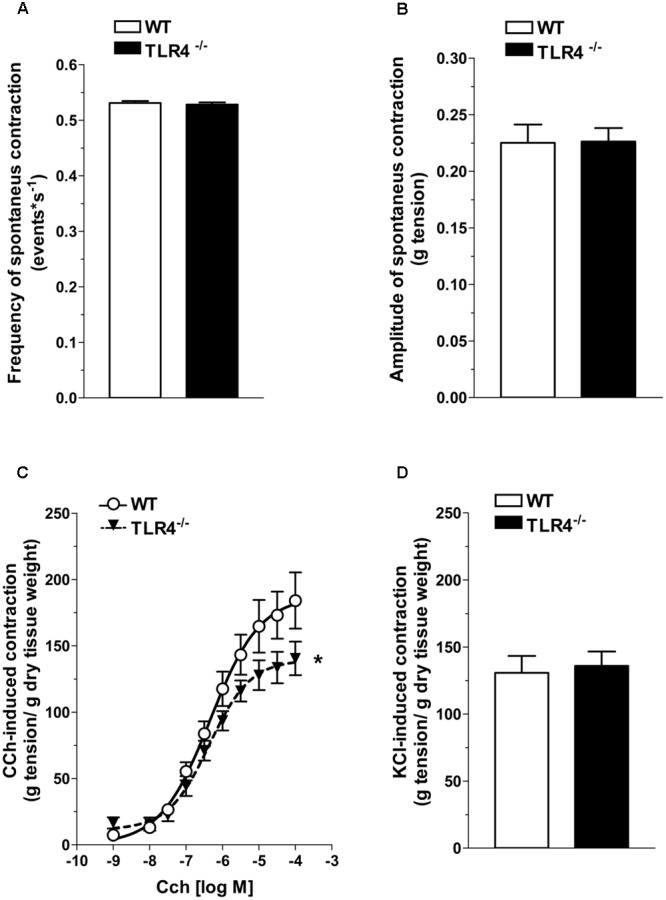
TLR4 deficiency impairs contractile responses to carbachol but not to high KCl. Frequency **(A)** and amplitude **(B)** of spontaneous contraction in WT and TLR4^-/-^ ileal preparations (*N* = 15 mice/group). Concentration–response curves to carbachol (CCh, **C**) and KCl-mediated excitatory response **(D)** in WT and TLR4^-/-^ preparations (*N* = 8 mice/group). ^∗^*P* < 0.05 vs. WT.

Based on the delayed gastrointestinal transit, we assessed spontaneous contractility that resulted comparable in frequency and amplitude in both genotypes (**Figures 3A,B**). To evaluate excitatory responses, cumulative concentration-response curves to the non-selective cholinergic receptor agonist, carbachol (CCh) were performed. Ileal segments from TLR4^-/-^ mice showed a significant downward shift of the concentration-response curve to CCh and a significant related decrease in the maximum response compared to WT (*E*_max_ = -25.6 ± 7.5%; **Figure 3C**). However, the response to high potassium-induced depolarization was similar in both genotypes (**Figure 3D**).

**FIGURE 4:**
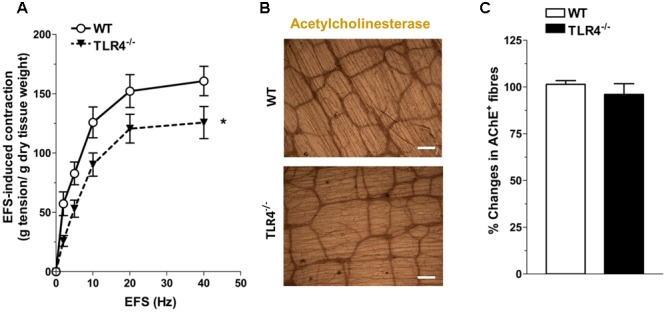
TLR4 deficiency alters ileal excitatory contractility. **(A)** EFS-induced excitatory responses in WT and TLR4^-/-^ preparations (*N* = 8 mice/group). Representative microphotographs showing the distribution **(B)** and % changes **(C)** of AChE^+^ fibers in WT and TLR4^-/-^ preparations (*N* = 5 mice/group). Bars = 200 μm. ^∗^*P* < 0.05 vs. WT.

Since ENS structural abnormalities have been described in *Tlr4^Lps-d^* mice (Anitha et al., 2012), we sought to test neuromuscular function by analyzing frequency-response curves to EFS. Altered neurotransmission in TLR4^-/-^ ileal segments was reflected by reduced EFS-elicited contractions (by 28.5 ± 9.7% at 10 Hz, **Figure 4A**). The EFS-induced contractions up to 10 Hz were of neuronal cholinergic origin as confirmed by their sensitivity to tetrodotoxin (TTX) and to the muscarinic receptor blocker atropine, as previously shown (Brun et al., 2013). However, no changes in the number of AChE^+^ fibers were found in TLR4^-/-^ whole-mount preparations (**Figures 4B,C**).

**FIGURE 5:**
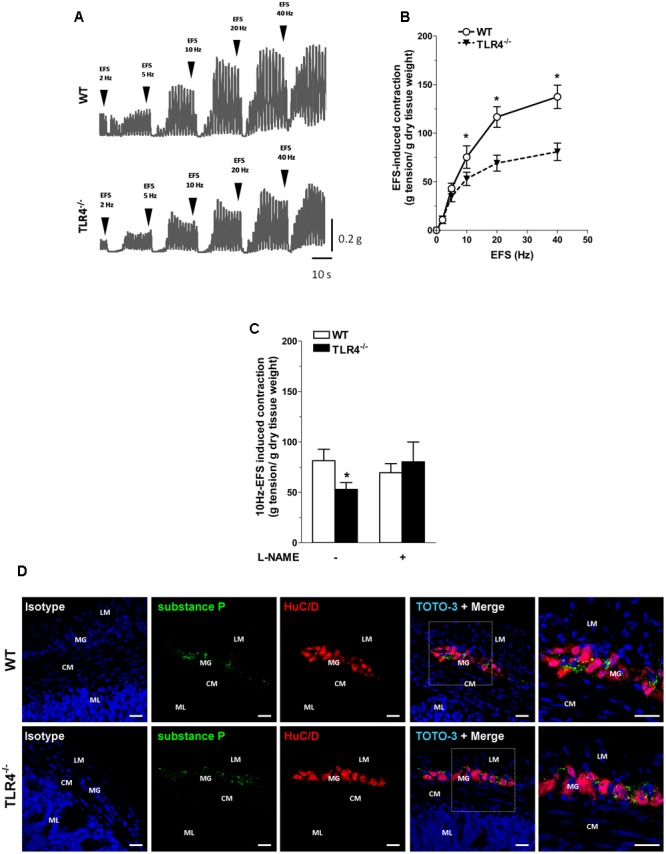
Tachykininergic neurotransmission is not affected by TLR4 deficiency. **(A)** Representative traces of contractile responses to increasing EFS frequencies in WT and TLR4^-/-^ segments under NANC conditions. **(B)** NANC responses evoked by EFS. **(C)** Tachykininergic nerve-evoked contractions induced by 10 Hz-EFS, in NANC condition with or without L-NAME in WT and TLR4^-/-^ preparations (*N* = 8 mice/group). **(D)** Representative confocal microphotographs of HuC/D (red) and substance P (green) distribution in WT and TLR4^-/-^ cryosections. Cell nuclei were stained with TOTO-3 (blue; *N* = 5 mice/group). Bars = 22 μm. LM, longitudinal muscle; CM, circular muscle; MG, myenteric ganglia; ML, mucosal layer. ^∗^*P* < 0.05 vs. WT.

To evaluate the contribution of other excitatory neurotransmitters besides acetylcholine, we evaluated the post-stimulus excitatory responses in NANC conditions, which are determined by tachykininergic neurotransmission (Lecci, 2002). In WT mice, NANC responses evoked by EFS determined a transient relaxation of ileal preparations, followed by TTX-sensitive excitatory responses (**Figures 5A,B**) (Zizzo et al., 2003). These excitatory responses were significantly reduced in TLR4^-/-^ ileal segments (by 30 ± 8.3%; **Figures 5A,B**). Upon addition of L-NAME, a tachykinin-mediated excitatory response (Lecci, 2002) was found in TLR4^-/-^ preparations comparable to WT (**Figure 5C**). Accordingly, no differences were observed in the immunofluorescence distribution of substance P (SP) in WT and TLR4^-/-^ frozen sections (**Figure 5D**).

### TLR4 Modulates Inhibitory Neurotransmission

**FIGURE 6:**
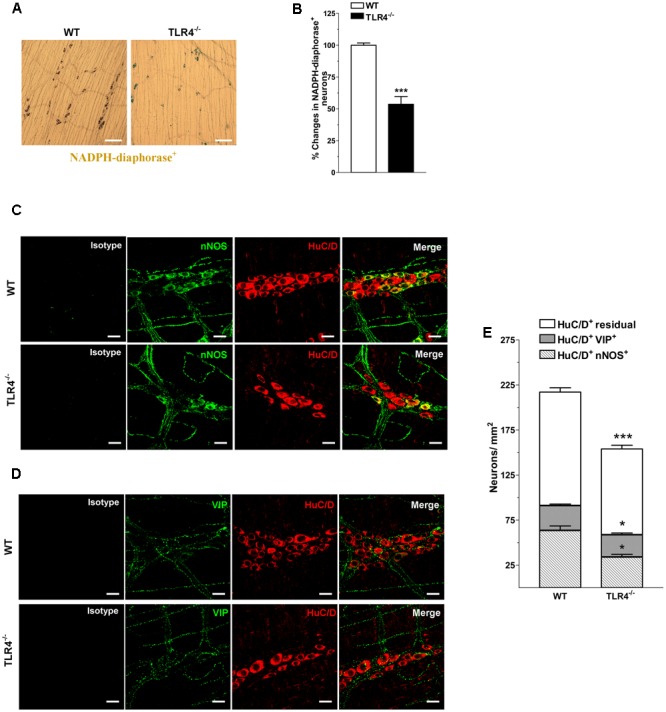
TLR4 signaling modulates nitrergic and VIPergic neurotransmissions. Representative microphotographs showing the distribution **(A)** and density **(B)** of NADPHd^+^ neurons in WT and TLR4^-/-^ LMMP preparations (bars = 300 μm). Representative confocal microphotographs showing the distribution of nNOS (**C**; green), VIP (**D**; green) and HuC/D (**C,D**; red) in WT and TLR4^-/-^ LMMP preparations (bars = 22 μm). **(E)** Number of HuC/D^+^nNOS^+^, HuC/D^+^VIP^+^, and residual HuC/D^+^ neurons in WT and TLR4^-/-^ LMMP preparations (*N* = 8 mice/group). *^∗^P* < 0.05, *^∗∗∗^P* < 0.001 vs. WT.

Considering that nitrergic neurotransmission (Zizzo et al., 2004; Lomax et al., 2010), the primary inhibitory pathway in the gut, is affected by dysbiosis (Kabouridis and Pachnis, 2015), we tested whether the reduced excitatory contraction could be the result of an increase of the inhibitory component. Consistent with this prediction, a reduction of nitrergic neurons stained with NADPH diaphorase (NADPHd) or anti-nNOS was observed in TLR4^-/-^ preparations (**Figure 6**). These changes were accompanied by a significant reduction of the total number of HuC/D^+^ neurons in TLR4^-/-^ myenteric plexus (**Figures 6C–E**). The reduction in nNOS^+^ neurons was associated with a proportional increase of VIP^+^ neurons in TLR4^-/-^ mice (**Figure 6**).

**FIGURE 7:**
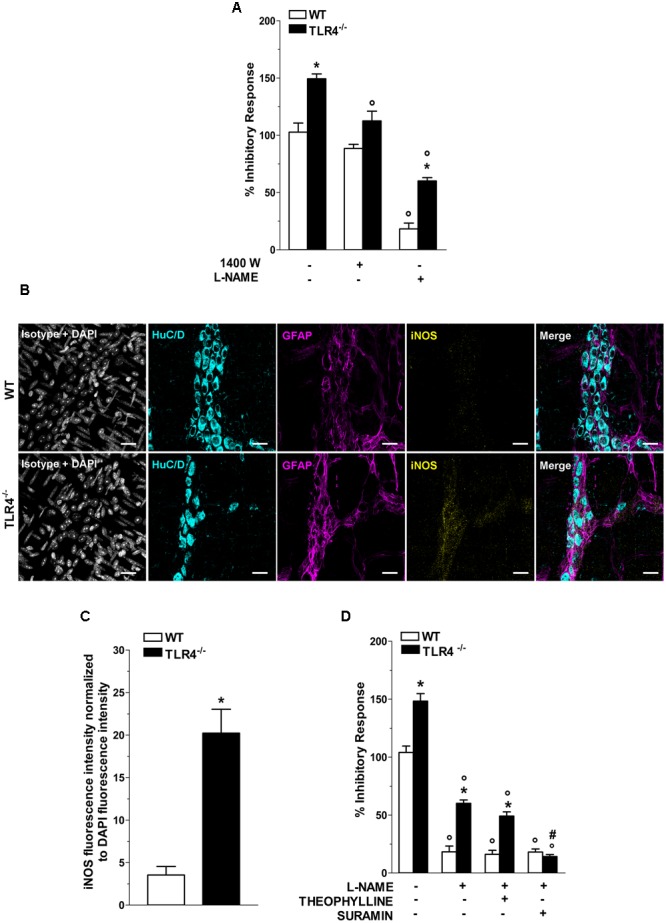
TLR4 signaling modulates NO and P2 receptor-mediated relaxation. **(A)** 10 Hz-EFS-evoked NANC relaxation responses with or without 1400 W or L-NAME in WT and TLR4^-/-^ preparations (*N* = 8 mice/group). **(B)** Representative confocal microphotographs showing HuC/D (cyan), GFAP (magenta) and iNOS (yellow) distribution (bars = 22 μm) and **(C)** analysis of changes in iNOS fluorescence intensity in WT and TLR4^-/-^ LMMP preparations (*N* = 5 mice/group). **(D)** 10 Hz-EFS-evoked NANC relaxation responses with or without L-NAME+theophylline or L-NAME+suramin in WT and TLR4^-/-^ preparations (*N* = 8 mice/group).^∗^*P* < 0.05 vs. WT; °*P* < 0.05 vs. respective control without L-NAME; ^#^*P* < 0.001 vs. respective control with L-NAME.

In NANC conditions EFS at 10 Hz caused a 1.48-fold increase in relaxation in TLR4^-/-^ mice (**Figure 7A**). Pretreatment with 1400W, a selective inhibitor of iNOS, significantly reduced the NANC-mediated relaxation in TLR4^-/-^ mice (by 25.2 ± 0.5%, **Figure 7A**), whereas a slight but not significant relaxation (13.8 ± 0.9%, **Figure 7A**) was recorded in WT. These findings support an involvement of iNOS in NO-mediated relaxation in the absence of TLR4. Furthermore, iNOS immunoreactivity increased by 5.7-fold in TLR4^-/-^ myenteric neurons and EGCs compared to WT (**Figures 7B,C**). Pretreatment with the pan-NOS inhibitor L-NAME almost completely blocked EFS-evoked NANC relaxation in WT mice. Conversely, in TLR4^-/-^ mice, this response was only partially abolished by L-NAME (**Figure 7A**) suggesting an influence of TLR4 in nitrergic-mediated relaxation and possibly in other inhibitory pathways (e.g., purinergic or VIPergic), known to sustain intestinal contractility (Zizzo et al., 2004). Accordingly, we evaluated the role of adenosine and adenosine-5′-triphosphate (ATP) in modulating relaxation in NANC conditions. Pretreatment with L-NAME and theophylline, a non-selective adenosine receptor antagonist, partially reduced the amplitudes of NANC-mediated relaxation in both genotypes whereas the addition of L-NAME and suramin, a non-selective ATP receptor antagonist, resulted in a significant reduction of the inhibitory response (by 75.3 ± 1.5%, **Figure 7D**) in TLR4^-/-^ mice, reaching a relaxation amplitude comparable to WT.

### TLR4 Absence Affects Purinergic Inhibitory Neurotransmission

**FIGURE 8:**
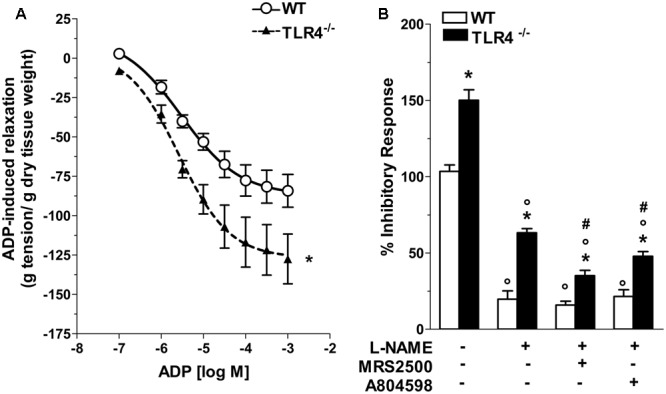
Involvement of TLR4 signaling in purinergic neurotransmission. **(A)** Concentration-response curve to ADP in WT and TLR4^-/-^ preparations (*N* = 8 mice/group). **(B)** 10 Hz-EFS-evoked NANC relaxation responses with or without L-NAME+MRS2500 (a P2Y1Rs antagonist) or L-NAME+A804598 (a P2X7Rs antagonist) in WT and TLR4^-/-^ preparations (*N* = 8 mice/group).^∗^*P* < 0.05 vs. WT; °*P* < 0.05 vs. respective control without L-NAME; ^#^*P* < 0.001 vs. respective control with L-NAME.

Considering that inhibitory neurotransmission in TLR4^-/-^ mice depends on both nitrergic and ATP-mediated relaxation, we evaluated the modulatory effect of ADP, the endogenous agonist of P2Y1 receptors (P2Y1Rs). TLR4^-/-^ mice showed a 1.43-fold increase in relaxation amplitude following addition of ADP in the organ bath with a significant shift to the left of the dose-response curve to ADP compared to WT mice (**Figure 8A**). Since also enteric P2X7 receptors (P2X7Rs) respond to ATP by mediating inhibitory neurotransmission and are involved in neuronal death during intestinal inflammation (Antonioli et al., 2014; Brown et al., 2015), we examined the influence of P2Y1Rs and P2X7Rs in NANC-mediated relaxation in the absence of TLR4. In ileal tissues from TLR4^-/-^ mice, P2Y1Rs blockade with MRS2500 in presence of L-NAME markedly reduced the amplitudes of NANC-mediated relaxation (by 45.1 ± 3%) whereas the addition of A804598 (a selective P2X7Rs antagonist) determined a reduction of the inhibitory response (by 24.2 ± 1.5%, **Figure 8B**), suggesting an involvement of both receptors in modulating relaxation in NANC conditions in the absence of TLR4.

**FIGURE 9:**
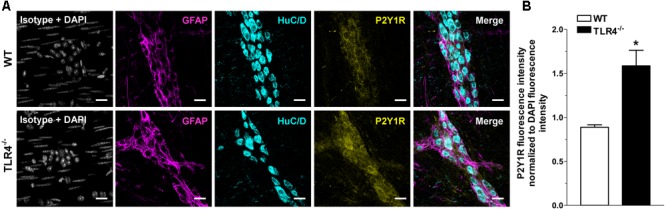
TLR4 signaling influences P2Y1 receptor distribution. Representative confocal microphotographs showing **(A)** GFAP (magenta), P2Y1Rs (yellow) and HuC/D (cyan) distribution and **(B)** analysis of P2Y1Rs fluorescence intensities in WT and TLR4^-/-^ LMMP preparations (*N* = 5 mice/group). Cell nuclei were stained with DAPI (gray). Bars = 22 μm. ^∗^*P* < 0.05 vs. WT.

**FIGURE 10:**
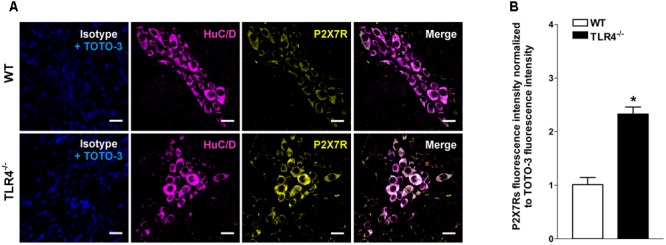
TLR4 signaling influences P2X7 receptor distribution. Representative confocal microphotographs showing **(A)** HuC/D (magenta) and P2X7Rs (yellow) distribution and **(B)** analysis of P2X7Rs fluorescence intensities in WT and TLR4^-/-^ preparations (*N* = 5 mice/group). Cell nuclei were stained with TOTO-3 (blue). Bars = 22 μm. ^∗^*P* < 0.05 vs. WT.

Immunohistochemical analysis revealed a 1.79-fold increase of P2Y1Rs staining in myenteric ganglia, in both neurons and EGCs (**Figure 9**) and a 2.4-fold increase in P2X7Rs immunoreactivity in TLR4^-/-^ myenteric neurons, underlining the involvement of TLR4 in ensuring ENS homeostasis (**Figure 10**).

## Discussion

TLR4 plays a well-established regulatory role in the innate immune response to infection and in adaptive responses consenting probiotic bacteria colonization. To date, several studies have evaluated the role of TLR4 in gut mucosa, mainly in colon, whereas only few have explored the impact of TLR4 in the ENS of small intestine (Anitha et al., 2012; Kabouridis and Pachnis, 2015).

Although polymorphisms in TLR4 gene affecting LPS signaling have been described in patients with chronic inflammatory bowel diseases (IBD), their pathophysiological relevance in neuroimmune cross talk is still unclear (Cario, 2010). In this regard, ENS appears to be directly involved in modulating the inflammatory response, since it express several TLRs, including TLR4, in enteric neurons and glial cells (Rumio et al., 2006; Sharkey and Savidge, 2014). In human longitudinal muscle-myenteric plexus (LMMP) and in rat ENS primary cultures, neural activation with EFS or ATP has been shown to inhibit LPS-induced TNF-α production through enteric neuronal P2X7R (Coquenlorge et al., 2014). However, it is becoming clear that TLR4 overstimulation by periodic intestinal infections, or its understimulation following excessive use of antibiotics have the potential to affect the balance between ENS-microbial-derived products early in life setting the basis for developing GFD in adulthood (Becattini et al., 2016).

Here, for the first time, we show the role of TLR4 on ENS structural and functional integrity and provide relevant insights into the underlying mechanisms. In particular, this study demonstrates that the absence of TLR4 results in: (i) altered distribution of the enteric glial markers GFAP and S100β; (ii) decreased total number of HuC/D^+^ neurons; (iii) an altered nNOS^+^-to-VIP^+^ neuron ratio; (iv) impaired tonic cholinergic excitation; (v) enhanced inhibitory neurotransmission mediated by the coordinated action of both NO (from nNOS and iNOS) and ATP through the interaction with the purinergic P2X7Rs and P2Y1Rs.

Previous immunohistochemical analyses have shown the presence of TLR4 in gut neuromuscular layers, as well as in sensory dorsal root ganglia, suggesting that both intrinsic and extrinsic neuronal circuits possess the machinery to respond directly to microbiota-derived stimuli (Barajon et al., 2009). Indeed, our study showed that TLR4 deficiency does affect the distribution of S100β and GFAP, specific markers for EGCs (Gulbransen and Sharkey, 2012; Neunlist et al., 2014). Recently, the role of EGCs has started to emerge not only as a mechanical support for enteric neurons but as cellular integrative bridge of gut homeostasis involved in controlling neuroplasticity, mucosal barrier and inflammatory responses by releasing specific gliomediators (e.g., NO, ATP) (Gulbransen and Sharkey, 2012; Neunlist et al., 2014). Considering that the pathophysiological functions of GFAP and S100β in the ENS are still under discussion and that TLR4^-/-^ mice showed no changes in mucosal permeability and inflammatory markers (as shown by us and others) (Peterson et al., 2010; Devaraj et al., 2011), the increases in these regulatory and structural proteins advocate for the presence of an underlying gliopathy in absence of TLR4 signaling. In support of this premise, increases in GFAP expression are associated with EGCs differentiation, inflammation, and injury (Ochoa-Cortes et al., 2016); S100β expression and release by ECGs at μM levels are linked to pathological conditions (Rühl, 2005); and EGCs gliosis, detected by increased GFAP levels and/or S100β immunoreactivity/release, has been reported in ulcerative colitis, microbial infection and neurodegenerative diseases (Ochoa-Cortes et al., 2016).

Lipopolysaccharide hyporesponsiveness and delayed gastrointestinal transit were reported for *Tlr4^Lps-d^* mice on a C3H/HeJ background. However, our study was performed at earlier time points (30 min vs. 4 h) following non-absorbable FITC-dextran administration to evaluate the involvement of the small intestine in the delayed transit. Although the reduced pellet frequency and water content may resemble the same colon dysmotility previously described in *Tlr4^Lps-d^* mice (Anitha et al., 2012), our mouse model has a spontaneous mutation that results in a complete loss-of-function of TLR4, whereas the *Tlr4^Lps-d^* mice have a point mutation causing an amino acid substitution. In our study, no differences in high potassium-induced contraction were revealed, suggesting that TLR4 deficiency does not influence smooth muscle function (Ratz et al., 2005). However, the downward shift of the concentration-response curve to both carbachol and EFS in TLR4^-/-^ preparations may be explained by the different contributing triggers to the onset of the reduced excitatory neuromuscular response, such as impaired cholinergic neurotransmission, higher level of inflammatory mediators or enhanced inhibitory non-adrenergic non-cholinergic transmission.

By expressing TLR4 and thus being sensitive to LPS, enteric neurons appear to be involved in the regulation of the innate tolerance response to microbial-derived products in the intestine, ensuring a balanced immune response with respect to luminal content. Both increased and decreased GI motility have been reported after LPS exposure, depending on the dose, timing between injection and assessments of GI motility, the region of the GI system that is investigated and the type of LPS (Bashashati et al., 2012). Low-grade inflammation in the gut can alter digestive motility, through changes in the functions of enteric nerves and/or smooth muscle cells, thus highlighting a pathophysiological relationship between bowel inflammation and abnormalities in enteric motor activity (Kabouridis and Pachnis, 2015). However, recent studies have detected no differences in the levels of several inflammatory markers measured in serum and in peritoneal macrophages in TLR4^-/-^ animals compared to C57BL/6J mice (Hritz et al., 2008; Zhang et al., 2008; Devaraj et al., 2011).

Alterations in tachykininergic pathways have been shown in GFD (Margolis and Gershon, 2009; Corsetti et al., 2015). Under NANC conditions, post-stimulus excitatory responses with L-NAME showed no differences between genotypes, consistent with a lack of TLR4 involvement in tachykininergic pathways also confirmed by immunohistochemistry of SP.

Since under certain conditions cholinergic nerve activity can be depressed (Anitha et al., 2006), the observed marked NANC-mediated relaxation indicates that impaired cholinergic neurotransmission results from an enhanced inhibitory control on cholinergic and noradrenergic transmission. The main inhibitory neurotransmitter NO can be generated by the three different enzymes, nNOS, eNOS, and iNOS. More than 90% of the total NOS in the small intestine is nNOS, localized in inhibitory neurons. However, iNOS isoform is also constitutively present and accounts for less than 10% of the total enteric NOS activity whereas eNOS isoform is barely detectable (Lu et al., 2006). In case of inflammation the induction of iNOS produces a large amount of NO with consequent intestinal dysmotility (Eskandari et al., 1999). NANC-mediated relaxation was increased in TLR4^-/-^ preparations and was mediated by NO produced by iNOS and nNOS, whereas in WT mice the inhibitory tone was mainly dependent on nNOS-derived NO. Moreover, TLR4^-/-^ myenteric ganglia contained a reduced number of HuC/D^+^ neurons, associated to a proportional reduction of nNOS^+^ neurons in agreement with our functional findings and as previously shown by Anitha et al. (2012) in whole mount preparations. However, we found that this reduction in nNOS^+^ neurons was accompanied with a proportional increase of VIP^+^ neurons. VIP not only acts as a neurotransmitter but also plays a role in neuroprotection and functions as an anti-inflammatory agent (Ekblad and Bauer, 2004). These adaptive changes in the proportion of VIP^+^ and nNOS^+^ neurons with no modifications in SP-containing nerves appear to be phenotypic characteristics of ENS resembling those found in diabetic neuropathy (Voukali et al., 2011). At the functional level, the loss of TLR4 appears to influence the nitrergic pathway engaging other inhibitory transmitters responsible for the increased NANC relaxations. Our findings of gliosis and enhanced inhibitory tone, sensitive to both L-NAME and suramin (a non-selective P2 receptor antagonist), support the involvement of ATP in the ENS dysfunctions of TLR4^-/-^ mice.

ATP is known to play important roles in gut function as well as in inflammation, since its P2 receptors are widely distributed in neurons, glia, smooth muscle, and immune cells (Burnstock, 2014). In mouse ileum, pharmacological studies (Giaroni, 2002; Gallego et al., 2014) have shown that in physiological conditions ATP modulates relaxation by acting via P2Y1Rs, the main receptor subtype mediating NANC inhibitory responses, partly by direct action on smooth muscle and partly by activating nNOS^+^ neurons that release ATP and NO. In the presence of inflammation, a overproduction of ATP activates the low affinity P2X7Rs, contributing significantly to activating inhibitory nitrergic neurons (Hu et al., 2001; Antonioli et al., 2014). In this respect, it has been proposed that ATP released by enteric neurons can activate EGCs, which in turn through Ca^2+^ signals and release of gliotransmitters (e.g., ATP, glutamate, among others), communicate with other ECGs and neurons, influencing gut contractility (Ochoa-Cortes et al., 2016). Here, we found higher amplitude of relaxation to ADP (the P2Y1Rs endogenous ligand) and NANC-mediated responses sensitive to P2Y1 and P2X7 inhibition, together with an increase in immunoreactivities of P2Y1Rs, P2X7Rs and iNOS in TLR4^-/-^ myenteric plexus. Our functional results support the notion that TLR4 deficiency influences ATP neurotransmission, activating myenteric P2X7Rs and P2Y1Rs and determining increased smooth muscle relaxation and iNOS-derived NO production potentially by enteric neurons and EGCs.

Recent studies so far have shown the involvement of TLR4 in the modulation of enteric neural stem/progenitor cells, and of neural survival (Anitha et al., 2012; Schuster et al., 2014), our work provides the first evidence of a cross-talk between TLR4 and nitrergic/purinergic pathways in enteric neural-glial communication (Rumio et al., 2006; Brown et al., 2015). Specifically, our study advocates for a new scenario in the ENS, where in the absence of TLR4, ATP pathways cooperate with nitrergic neurotransmission through P2Y1Rs and P2X7Rs as recently demonstrated by Brown et al. (2015).

## Conclusion

Our study highlights for the first time a novel role for TLR4 in ileum, demonstrating that TLR4 fine-tunes ENS circuitry modulating the inhibitory component of neuromotor activity by NO and ATP co-transmission, essential for maintaining a proper bidirectional neural-glial communication. Our findings provides the basis for a better understanding of the mechanisms underlying gastrointestinal dysmotility in presence of an anomalous neuroimmune cross talk, thereby paving the way for the development of suitable pharmacological modulators of TLR4 signaling for the management of GFD.

## Author Contributions

Conceived and designed the experiments: MG, CG, GO, VC, IM, and EN. Performed the experiments: VC, IM, MoR, SC, and FG. Analyzed the data: VC, IM, GO, FG, EN, MaR, and IL. Contributed reagents/materials/analysis tools: MG, GO, IL, and MaR. Wrote the manuscript: MG, VC, IM, CG, and EN. All the authors reviewed the manuscript.

## Conflict of Interest Statement

The authors declare that the research was conducted in the absence of any commercial or financial relationships that could be construed as a potential conflict of interest.
